# Longitudinal associations between infant movement behaviours and development

**DOI:** 10.1186/s12966-022-01248-6

**Published:** 2022-01-28

**Authors:** Valerie Carson, Zhiguang Zhang, Madison Predy, Lesley Pritchard, Kylie D. Hesketh

**Affiliations:** 1 Faculty of Kinesiology, Sport, and Recreation, University of Alberta, Edmonton, AB Canada; 2 Faculty of Rehabilitation Medicine, University of Alberta, Edmonton, AB Canada; 3 Institute for Physical Activity and Nutrition, Deakin University, Geelong, Vic Australia

**Keywords:** Infants, Tummy time, Sedentary behaviour, Sleep, Development, Longitudinal

## Abstract

**Background:**

This study aimed to address methodological limitations of the evidence that informed national and international movement behaviour guidelines for the early years. Specifically, the primary objective was to examine the longitudinal associations of infant physical activity (i.e., tummy time) and sedentary behaviour (i.e., back time, screen time, reading time, restrained time) with gross motor development. Secondary and tertiary objectives were to examine longitudinal associations of: (1) infant physical activity and sedentary behaviour with communication, fine motor, personal-social, and problem solving development, and (2) sleep time with primary and secondary outcomes.

**Methods:**

Participants were 411 parents and their infants from the Early Movers project in Edmonton, Canada. Physical activity, sedentary behaviour, and sleep were measured with a parental questionnaire and the Ages & Stages Questionnaire (ASQ-3) developmental screening tool was administered at 2, 4, and 6 months. Parents reported the dates six major gross motor milestones (i.e., independent sitting, crawling, assisted standing, assisted walking, independent standing, independent walking) were acquired in the first 18 months of life according to World Health Organization criteria. In a subsample (*n* = 125), gross motor development was assessed using the Alberta Infant Motor Scale (AIMS) at 6 months.

**Results:**

Higher tummy time across time points was significantly associated with higher ASQ-3 gross motor and personal-social development scores over time, higher total AIMS scores at 6 months, and earlier acquisition of all gross motor milestones. Higher reading time across time points was significantly associated with higher ASQ-3 fine motor, gross motor, personal-social, and total development scores over time. In contrast, higher back time across time points was significantly associated with lower total AIMS scores at 6 months and the later acquisition of assisted standing, assisted walking, and independent walking. Similarly, higher restrained time across time points was significantly associated with a later acquisition of supported walking.

**Conclusions:**

Tummy time was consistently longitudinally associated with more advanced gross motor development and reading with more advanced total development. Whereas, some detrimental associations were observed for back and restrained time. Findings support the promotion of tummy time and certain sedentary behaviours (i.e., reading) in young infants to enhance overall development.

**Supplementary Information:**

The online version contains supplementary material available at 10.1186/s12966-022-01248-6.

## Background

To support the promotion of healthy physical, social-emotional, and cognitive development, new Canadian 24-Hour Movement Guidelines for the early years were released in 2017. These guidelines include physical activity, sedentary behaviour, and sleep recommendations for infants (< 1 year), toddlers (1–2 years), and preschoolers (3–4 years) to provide guidance for policymakers, relevant health professionals, public health practitioners, early childhood educators, parents, and caregivers [1]. Other countries and the World Health Organization (WHO) have adopted the Canadian guidelines [2], which are based on the best available evidence, expert consensus, and stakeholder input [1].

One important outcome of guideline development is the identification of key evidence gaps. Identified gaps can stimulate future research that informs updates to the guidelines, health promotion campaigns, interventions, and policies to improve the health of a population [1, 2]. In the physical activity and sedentary behaviour systematic reviews that were used to inform the national and international guidelines, similar evidence gaps were apparent regarding typically developing children [3, 4]. Specifically, evidence was overwhelmingly limited to cross-sectional studies (only 13 and 26% of included studies for physical activity and sedentary behaviour, respectively, were longitudinal) and most studies focused on preschool children (70 and 84% of included papers for physical activity and sedentary behaviour, respectively), with limited evidence on infants. Evidence was also disproportionally weighted to associations with adiposity (60 and 65% of included papers for physical activity and sedentary behaviour, respectively), compared to other important health indicators, such as motor, social-emotional, and cognitive development [3, 4]. Given these systematic reviews focused on studies with a minimum sample of 100 participants for observational designs, longitudinal evidence on the association between physical activity, sedentary behaviour, and development among infants from population-based samples are particularly lacking [3, 4].

For infants who are not yet mobile (e.g., crawling), an important type of physical activity is supervised time on the tummy or in the prone position while awake [3]. In the previously mentioned physical activity systematic review [3], only three studies were found examining the association between tummy time and development in infants [5–7]. Though all three studies reported an association between more tummy time and more advanced motor development, these studies had limitations [3], including the use of a cross-sectional design [5, 6], convenience samples [6], questionnaires with unknown psychometric properties [5–7], and unadjusted analyses [6]. Another systematic review specifically focusing on infant tummy time and health indicators, with a smaller sample size criterion for observational studies (≥30 participants), was conducted after the release of the Canadian guidelines [8]. Similarly, it was reported that more tummy time was consistently associated with more advanced motor development, including the earlier acquisition of some gross motor milestones as well as more advanced total development [8]. However, limited evidence was found for other domains of development and consistent with the previous systematic review [3], methodological limitations existed among included studies [8]. In particular, all studies included in the review were at risk for selection bias and high-performance bias related to the tummy time measure [8].

Sedentary behaviour among infants is a waking behaviour that involves low energy expenditure when restrained (e.g., in a carrier, car seat, stroller) or when sedate (e.g., lying on back or supine with minimal movement when not restrained) [4]. Being restrained is obviously a safety requirement in several situations for infants. However, prolonged time regularly spent in situations that restrict movement can displace the time infants have to play and develop gross motor skills [9]. Only one included study in the previously mentioned sedentary behaviour review examined the associations between restrained time and gross motor milestone attainment, and cross-sectional findings at 4, 9, and 20 months were primarily null [10]. An older systematic review on the associations between infant equipment, such as infant sitting devices, and motor development, which had no sample size inclusion criteria, reported inconsistent findings [9]. The low methodological quality of included studies and the need for longitudinal studies was noted in that review [9]. Other forms of sedentary behaviour for infants include screen time and non-screen based sedentary time (e.g., storybook reading) [11]. Of the limited included studies on screen time and reading in the systematic review that informed national guidelines, findings for screen time were also inconsistent, though more reading/storytelling was consistently associated with better cognitive development in infants [4].

The Early Movers project was specifically designed to address evidence gaps regarding the associations between physical activity, sedentary behaviour, and development among infants. The primary objective of this study was to examine the longitudinal associations of infant physical activity (i.e., tummy time) and different types of sedentary behaviour (i.e., back time, restrained time screen time, reading time) with gross motor development. The secondary objective was to examine the longitudinal associations of infant physical activity and sedentary behaviour with communication, fine motor, personal-social, and problem solving development. The tertiary objective was to examine the longitudinal associations between sleep time and primary (i.e., gross motor development) and secondary outcomes (i.e., communication, fine motor, personal-social, and problem solving development). In terms of the primary objective, it was hypothesized that: 1) longer duration of tummy time will lead to higher gross motor development scores and earlier achievement of gross motor milestones and 2) longer duration of restrained time will lead to lower scores of gross motor development and later achievement of gross motor milestones.

## Methods

### Study design and participants

The Early Movers project used a longitudinal study design to address research objectives and test hypotheses. In partnership with Alberta Health Services, parents/guardians (parents thereafter) and their infants were recruited between March 2018 and November 2019 from one of five Public Health Centres in Edmonton, Canada while attending a routine 2-month immunization appointment. Approximately 85% of the annual Edmonton birth cohort attends a health centre for their 2-month immunization (Personal Communication, AHS, May 8, 2017). The five public health centres were selected because they serve large communities with diverse demographic characteristics. Having an infant aged 2 months 0 days through 2 months 30 days at baseline was the only inclusion criteria for families to participant in the project. Families were excluded from the project if a parent did not confidently speak or read English or if their infant: 1) regularly cared for by an adult other than their parent/guardian for a number of hours per week (e.g., attended full-time childcare), 2) was born preterm (i.e., gestational age < 37 weeks), 3) was born underweight (i.e., < 2500 g), or 4) had a medical condition or health complication since birth that could have an impact on their physical activity, sedentary behavior, or development.

**Fig. 1:**
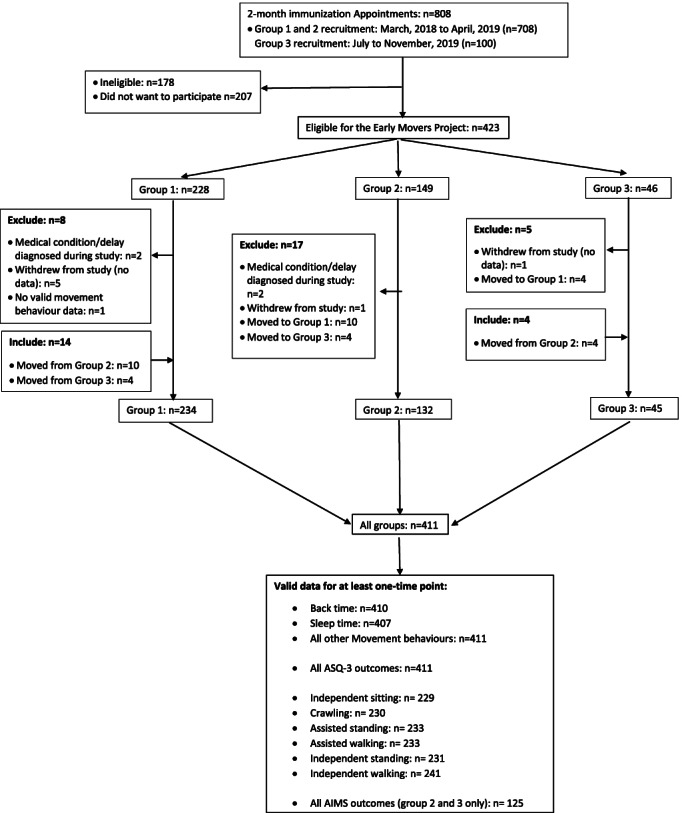
The Early Movers project recruitment flow chart. The Early Movers project was divided into three groups each with varying intensity of study measures. In all groups, parents were asked to complete a questionnaire at 2, 4, and 6 months and report gross motor milestones until 18 months. In group 2 and 3, parents were also asked to complete a time-use diary at 2, 4, and 6 months and infants’ gross motor development was assessed by a physiotherapist at 6 months of age. In group 3, parents were also asked to have their infant wear an accelerometer at 6 months. Abbreviation: ASQ, age and stage questionnaire; AIMS, Alberta Infant Motor Scale

Population-based studies often have a trade-off between the benefits of large and representative samples and measurement precision [12, 13]. These benefits and limitations were balanced by a group assessment structure employed in this study, with more precise and burdensome measures added to different groups. More specifically, participants in the Early Movers project were enrolled in one of three groups, each with varying intensity of study measures (see Fig. 1). All three groups participated in the main study, those in groups 2 and 3 also participated in a time-use diary sub-study, and those in group 3 also participated in a validity sub-study. Therefore, group 1 required the least amount of time to complete study measures (i.e., questionnaires, gross motor milestones), followed by group 2 (i.e., questionnaires, time-use diaries, directly-observed gross motor development, gross motor milestones) and group 3 (i.e., questionnaires, time-use diaries, accelerometer, directly-observed gross motor development, gross motor milestones). Between March, 2018 and April, 2019, participants were enrolled in group 1 (main study only) or group 2 (main study and time-use diary sub-study) based on parental choice. Near the end of the project, new evidence was published on the first valid device-based measure of tummy time in infants [14]. As a result, we conducted further recruitment between July and November, 2019 to enroll participants in group 3 (main study, time-use diary sub-study, and validity sub-study) with the aim of validating our questionnaire and time-use diary measures of tummy time against this device-based measure [15]. During this period of recruitment, groups 1 and 2 were not initially presented to parents. A total of 18 participants changed groups during the project to decrease the intensity of study measures (*n* = 14) or to add on the accelerometer measure (*n* = 4). The changing of groups did not have any implications on the timing or completion of measures in the new group. The present study included participants from all three groups as part of the main study. Ethics approval was obtained from the University of Alberta Research Ethics Board (Project # 00078438). Written informed consent was obtained from all participating parents.

Prior to study recruitment, a power calculation was conducted for the main study and the time-use diary sub-study and it showed that a sample size of 250 participants in the main study would be sufficient to detect a medium effect size (f2 = 0.15) in a linear regression with 12 variables including confounders, a power of 0.8, and a probability of 0.05 for a 2-tailed hypothesis [16, 17]. This calculation accounted for the design effect of time being nested in participants, using a conservative ICC of 0.1. It further accounted for a conservative rate of 20% loss to follow-up. Under the same parameters, 115 participants for the time-use diary sub-study would detect a medium to large effect size (f2 = 0.25). It was anticipated that with increased measurement precision, it would be possible to detect a slightly larger effect size. A sample size calculation was not conducted *apriori* for the validity sub-study but the sample size target was 50 for logistical reasons (i.e., timely completion of main study).

Between March, 2018 and January, 2019, the sample size target of 250 for the main study was reached but not for the time-use diary sub-study (*n* = 78) so recruitment continued. Participants could still choose between groups 1 and 2 in case loss to follow-up or missing outcome data was greater than anticipated for the main study. By April, 2019, we had reached our sample size target for the time-use diary sub-study. Recruitment for the validity sub-study between July and November, 2019, further increased sample size numbers for the main study and time-use diary sub-study.

### Procedures

Research staff visited the waiting room of public health centres during days and times when multiple 2-month immunization appointments were scheduled. Research staff discussed the study with parents before the appointment and/or after the appointment during the required 15-min wait period. If parents were eligible and interested in participating, they completed a consent form, a contact information form, and a parental questionnaire. Parents completed the information on a tablet using the secure web application REDCap [18] or on a hard paper copy. Research staff checked completeness to minimize missing data and ensure accurate contact information. At the appointment, participants were given a gross motor milestone booklet to take home to record the dates that six gross motor milestones were achieved. When infants turned 4 and 6 months of age, participants were e-mailed a survey link to follow-up parental questionnaires via REDCap. For participants who preferred a hard copy, questionnaires were sent and returned by mail. After the 6-month questionnaire, participants were contacted monthly to determine if and the date gross motor milestones were achieved. Monthly follow-ups occurred until the child had reached all the milestones or 18 months of age when 99% of children typically have acquired all six milestones [19].

At the immunization appointments between March, 2018 and April, 2019, families could choose to remain in group 1 (main study only) or enroll in group 2 (main study and time-use diary sub-sample). In addition to group 1 measures, participants in group 2 were asked to complete a 3-day/night time-use diary when the infant was 2, 4, and 6 months of age. Participants were encouraged to complete the time-use diaries throughout the day and were provided with a hard copy at the immunization appointment with verbal and written instructions. At 4 and 6 months, the time-use diaries were mailed to participants. Additionally, a physical therapist contacted families to schedule a home visit appointment to conduct a direct observation of infant’s gross motor development when they were between the ages of 6 months 0 days and 6 months 7 days. At the immunization appointments between July, 2018 and November, 2019, participants were enrolled into group 3 only (main study, time-use diary sub-study, and validity sub-study). In addition to group 2 measures, parents enrolled in group 3 were asked to have their infant wear an accelerometer on the waist at 6 months for 3 consecutive days. Further details regarding the time-use diary and accelerometer measures have previously been published [15]. Gift cards ranging from $10 to $50 CAD in value, depending on the group enrollment and the number of measures completed, were mailed to participants after completion of data collection.

### Measures

#### Movement behaviours

Infant movement behaviours were measured using the parental questionnaire when infants were 2, 4, and 6 months of age. Detailed instructions and examples were provided on how to record the typical daily hours and minutes of each movement behaviour. For tummy time and back time, parents were asked one question each about the average time their child spends awake on their stomach or back when they are free to move. For restrained time, parents were asked five separate questions regarding the average time their child spends awake in a: 1) stroller, 2) car seat, 3) baby carrier, sling, wrap, 4) indoor swing, and 5) high chair or other chair with safety straps. For reading time, parents were asked one question about the average time their child spends reading/looking at books with the parent or another child/adult. For screen time, parents were asked two separate questions regarding the average time their child spends: 1) watching/looking at the television and 2) watching/looking at a cell phone/tablet. Finally, for sleep time, parents were asked two separate questions regarding the average time their child: 1) usually sleeps in total per night at the moment (not including time spent feeding) and 2) usually naps in total during the day at the moment. For restrained time, screen time, and sleep time, responses were summed across questions. The majority of movement behaviour questions used in the Early Movers project were adapted from the Melbourne Infant Feeding Activity and Nutrition Trial (InFANT) Program. This study observed acceptable 2-week test-retest reliability for the majority of individual items when infants were 4 and 9 months of age [10]. In a validity study that included participants from group 3 (*n* = 26) of the Early Movers project, the questionnaire measure of tummy time was significantly correlated with a validated accelerometer measure of tummy time [14] with a large effect size (r_s_ = 0.60) when infants are 6 months of age [15, 16]. Lastly, among participants in groups 2 and 3 that had questionnaire and time-use diary data at each time point, significant correlations (r_s_ = 0.30–0.56) were observed between measures with medium to large effect sizes [16]. (see Supplementary File : Table S1).

#### Development

The primary outcome variables of interest were gross motor development measured at 2, 4, and 6 months of age and acquisition of gross motor milestones within the first 18 months of life. The secondary outcome variables were communication, fine motor, problem solving, and personal-social skills measured at 2, 4, and 6 months of age. Gross motor development and secondary outcome variables were measured with the Ages and Stages Questionnaire (ASQ-3), a commonly used parental-report developmental monitoring system that is suitable for diverse populations [20, 21]. At each time point, there were 30 age-specific items with three response options (i.e., yes, sometimes, not yet). Following ASQ-3 procedures, each participant was given a score between 0 and 60 for each outcome variable and a total score between 0 and 300 across all outcome variables, with a higher number indicating more advanced development [20]. When participants were missing 1 or 2 items for an outcome variable (*n* < 5 at each time point), scores for that outcome were adjusted (adjusted score = [original score/answered items] *6) [20]. Though, if ≥3 items were missing, that outcome variable was not scored and a total score was not calculated. The ASQ-3 was originally standardized using a sample of 15,128 children and has shown good criterion validity when compared to the Battelle Developmental Inventory-II [20]. For example, percent agreement was 100% at 2 months, 83.3% at 4 months, and 85.7% at 6 months [20]. The gross motor development scale specifically has shown moderate criterion validity (*r* = 0.46) with the Bayley Scales of Infant Development II [21].

The date children acquired six gross motor milestones including independent sitting, hands and knees crawling, assisted standing, assisted walking, independent standing, and independent walking, were reported by parents using the WHO caregiver assessment criteria [18]*.* Parents were provided with a booklet that included clear instructions and pictures developed by the WHO on how to assess the milestones [19]. The ages in days at which the child first achieved the milestones were calculated. Parents recorded whether the date provided was exact or approximate. In regards to approximate dates, if a parent reported a milestone occurred when the child was X months old, the milestone date was recorded as X months and 15 days. If a parent reported that a milestone was achieved early, mid, or late in a month, the 1st day, 15th day, or last day of the month was recorded, respectively. Lastly, if multiple dates were submitted for the same milestone and parents did not confirm the correct date, the earliest date was used. All exact and approximate milestone dates were reviewed by members of the research team, and the cleaning of milestone data followed 3 sequential rules. First, if the reported date was an outlier (i.e., < 1 percentile, > 99 percentile according to WHO percentiles [22]) and was approximate or did not align with other milestones, data for that milestone was removed (number of milestones removed =29 from 21 participants). Second if the reported date for any milestone occurred before or on the same day as independent sitting then data for the respective milestone and the independent sitting milestone were removed (number of milestones removed =38 from 17 participants). This decision was made because it was unlikely that any of the other motor milestones would occur before independent sitting based on the normative data from the WHO [22]. Third, if the reported dates for a pair of milestones occurred out of order based on WHO criteria and were over a month apart, data were removed for both milestones (number of milestones removed =8 from 4 participants). Hands and knees crawling was not considered out of order with any milestone unless reported before independent sitting.

Among participants in groups 2 and 3, gross motor development was also directly observed by a physical therapist using the Alberta Infant Motor Scale (AIMS) when infants were 6 months of age. The AIMS is a norm-referenced, 58-item observational measure of an infant’s gross motor development from birth to independent walking (0–18 months) [23]. The normative data set is based on approximately 2220 infants from Alberta in 1990–1992. The tool focuses on four postural positions: supine, prone, sitting, and standing, and takes into account three criteria for quality of movement including, weight distribution, posture, and movement against the force of gravity [23]. Following AIMS procedures, the four postural positions were given a score (Prone: 0–21, Supine: 0–9, Sitting: 0–12, Standing: 0–16) and a total score was calculated between 0 and 58. A percentile score (between 0 and 100) was assigned based on the infant’s age and total score. The tool is widely used internationally and has previously shown excellent one-week test-retest reliability (*r* = 0.96–0.99) and excellent concurrent validity with other commonly used motor development assessment tools (Bayley Scales of Infant Development motor scales: *r* = 0.98; Peabody Development Motor Scales: *r* = 0.97) [23].

#### Covariates

A number of infant and parental demographic characteristics were considered as covariates based on previous research [3, 4]. Infant age (days) and parental age (years) at each time point were calculated from the date of questionnaire completion and the birth dates reported in the 2-month parental questionnaire. Mean imputation was performed for missing parental age data at baseline for 5 participants. Infant sex (male, female), infant race/ethnicity (Caucasian, other), number of siblings (zero, one, and ≥ 2), parental marital status (married/living common-law, not married/ living common-law), parental education (below bachelor level, bachelor’s degree, above bachelor level), and parental country of birth (Canada, other) were also reported in the 2-month parental questionnaire. Infant race/ethnicity, number of siblings, marital status, parental education categories described in this section were collapsed from the original scales due to frequency distributions. Finally, non-parental care time (hours per week) were parental-reported at each time point.

### Statistical analysis

Statistical analyses were performed using SPSS version 26.0 (SPSS Inc., Chicago, IL, USA). First, unlikely (e.g., 10 h/day of tummy time; tummy time: *n* = 4; back time: *n* = 4; restricted time: *n* = 2; screen time: *n* = 2; read time: *n* = 2; sleep time: *n* = 5) and impossible (sleep time: *n* = 1; i.e., 35 h) observations were removed. Additionally, two observations for sleep time were removed due to missing observations for nap (*n* = 1) or nighttime (*n* = 1) sleep. Next, descriptive statistics were calculated for demographic, exposure, and outcome variables at relevant time points. For movement behaviours and ASQ-3 outcomes, linear mixed models were employed to calculate marginal means at each time point. Specifically, time was included as fixed and repeated effects in the models.

To examine the longitudinal associations between each movement behaviour and each ASQ-3 outcome, linear mixed models were conducted using growth curve modeling. First, infant age was centred around 60 days for all time points then model building was used to identify the model of best fit for each outcome variable based on the likelihood ratio test and the Akaike’s Information Criterion (AIC). The first model included a random intercept for participant and subsequent models gradually increased in complexity by adding a fixed linear slope of infant age, a fixed quadratic slope of infant age, and/or a random linear slope of infant age. Once the best model fit for each outcome variable was identified, separate models were run for each combination of exposure and outcome variables to examine the associations between each movement behaviours and each ASQ-3 outcome. To determine, which covariates should be included in the models, the associations between each covariate and outcome variable were examined using linear mixed models (ASQ-3) and linear regression models (milestone; AIMS). For each covariate, the association(s) in regard to at least one outcome variable met the cut-off of *p* < 0.10 [24, 25]. Therefore, all covariates were included in all analytic models. To be included in each model, participants had to have at least one observation for each of the exposure, outcome, and covariate variables. Linear mixed models use maximum likelihood to handle missing data [26]. Assumptions for linear mixed models were checked through visual inspection of residuals and all assumptions were met. A sensitivity analysis was conducted that examined associations in those that had valid data at all three time points (i.e., complete case analysis). Findings from the sensitivity analysis were then compared with the findings from the main analysis (i.e., at least one observation).

To examine the longitudinal associations between each movement behaviour and each gross motor milestone and AIMS outcome, linear regression models were conducted. Logistic regression models were performed to examine the association between each movement behaviour and the AIMS stand outcome. Specifically, when the distributions of the model residuals were visually checked to confirm the linear regression model assumptions were met, a non-normal distribution was observed for the AIMS stand outcome variable. As a result, this variable was dichotomized (value = 1 [score = 2, reference group]; value = 0 [score > 2]). All other assumptions were met across models. Linear and generalized mixed models could not be used for these analyses because the outcome variables were only collected at a single time point. As result, an average was calculated for movement behaviours across time points to be included in the linear and logistic regression models. First, multiple imputation by fully conditional specification (maximum iterations = 1000) was conducted to create 10 imputed data sets and predicted mean matching model to impute missing movement behaviour variables at any time point [27]. Imputation was separately conducted for the participants with gross motor milestone data and those with AIMS data. Movement behaviours at all time-points and covariates were included in the imputation models. For participants with AIMS data, these outcome variables were also included in the imputation model. Gross motor milestone data were not included in the relevant implementation models as some gross motor milestone data were missing because they were invalid and therefore should not have been imputed. After imputation, average movement behaviours were calculated across time points. The unstandardized coefficient for the association between each movement behaviour and each gross motor milestone or AIMS outcome was a pooling result for all imputation data sets. All covariates were included in all models, with the exception of infant age, which was not included in gross motor milestone models because the outcome variables were an age. Sensitivity analyses was conducted that examined associations in those that had valid data across at all three time points (i.e., complete case analysis). Findings from the sensitivity analysis were then compared with the findings from the main analysis where missing movement behaviour data were imputed. An additional sensitivity analysis was conducted examining the longitudinal associations between movement behaviours and gross motor milestones in those where exact milestone dates were reported. Findings from the sensitivity analysis were then compared with the findings from the main analysis where exact and approximate milestone dates were included.

Movement behaviour variables, with the exception of sleep time, were expressed as 10 min/day within all models described in this section to make the interpretation of the regression coefficients more meaningful. Sleep time was expressed as hours/day. Specifically, for the linear mixed models the unstandardized beta coefficient can be interpreted as the pooled within- and between-individual differences in the ASQ-3 outcome variable for a one-unit increase (i.e., 10 min/day or 1 h/day for sleep) in the movement behaviour overtime. For the linear regression models, the unstandardized beta coefficient can be interpreted as the mean difference in the gross motor milestone or AIMS outcome variable for a one unit increase (i.e., 10 min/day or 1 h/day for sleep) of average movement behaviours across the three time-points. For the logistic regression model, the odds ratio can be interpreted as the likelihood of achieving a higher AIMS stand score, compared to a lower score, for a one unit increase (i.e., 10 min/day or 1 h/day for sleep) of average movement behaviours across the three time-points. Statistical significance was defined as *p* < 0.05 for all analyses.

## Results

Of the 808 families recruited for the project across groups, 178 were ineligible and 207 declined to participate, leaving a sample of 423 families. The 178 families were ineligible for the following reasons: preterm and/or underweight (*n* = 73), English language barrier (*n* = 52), infant older than 2 months at appointment (*n* = 44), medical condition (*n* = 8), and regularly cared for by another adult (*n* = 1). Among those eligible, the participation rate was 67%. The 207 families declined participation for the following reasons: not interested (*n* = 90), too busy (*n* = 54), left right after appointment/in a rush to leave (*n* = 24), unknown (*n* = 17), travelling/moving (*n* = 14), fussy infant at appointment (*n* = 8). Of the 423 eligible families that agreed to participate, 12 were excluded (medical condition or delay diagnosed during the study; *n* = 4; withdrew before providing any data: *n* = 7; no valid movement behaviour data at any time point: *n* = 1) leaving a sample of 411 participants (Group 1: *n* = 234, Group 2: *n* = 132, Group 3: *n* = 45; see Fig. 1).

Descriptive information on baseline demographic characteristics are presented in Table 1. Slightly more than half of infants were female (56%). Overall, the sample was relatively diverse. Specifically, just over half of infants identified as a race/ethnicity other than Caucasian (51%), approximately a third of parents were born in a country other than Canada (31%), and just under half of parents had an educational level below a bachelor’s degree (45%). Descriptive information on movement behaviours and development outcomes across relevant time points are presented in Table 2. One participant was missing valid back time data across time points and 4 participants were missing valid sleep time data across time points. Descriptively, marginal means for tummy time, reading time, and screen time increased from 2 to 6 months, whereas back time, restrained time, and sleep time decreased. ASQ-3 data were available in all 411 participants for at least one time point, and descriptively marginal means followed a curvilinear pattern for all variables. Valid milestone data were available from 229 to 244 participants, depending on the milestone. Marginal sample means were all close to the 50th percentile scores based on a WHO reference study of 816 healthy children from Ghana, India, Norway, Oman, and the United States [19]. Finally, 125 participants had valid AIMS data at 6 months.

**Table 1:** Baseline demographic characteristics

Characteristics	n	2 months
Infant age (days)	411	66.88 (6.12)
Infant sex		
Male	180	43.8
Female	231	56.2
Infant race/ethnicity		
Caucasian	203	49.4
Other	208	50.6
Number of siblings		
Zero	190	46.2
One	157	38.2
Two or more	64	15.6
Non-parental care time (hours)	411	2.26 (11.95)
Parental age (years)	411^a^	31.78 (5.32)
Parental marital status		
Married or living common-law	388	94.4
Not Married or living common-law	23	5.6
Parental education		
Above bachelor’s degree	83	20.2
Bachelor’s degree	142	34.5
Below bachelor’s degree	186	45.3
Parental country of birth		
Canada	283	68.9
Other country	128	31.1

^a^Mean imputation was performed for missing parental age at baseline (*n* = 5)

Data presented as mean (standard deviation) for continuous variables and percentages for categorical variables

**Table 2:** Movement behaviours and development outcomes

	2 months		4 months		6 months	
	n		n		n	
Tummy time (min/day) ^a^	411	47.91(2.91)	411	61.71(2.91)	411	116.31(5.99)
Back time (min/day) ^a^	410	178.02(7.90)	410	177.66(8.24)	410	127.00(6.98)
Restrained time (min/day) ^a^	411	191.01(6.95)	411	188.71(6.75)	411	182.46(6.29)
Reading time (min/day) ^a^	411	15.75(1.41)	411	23.10(1.26)	411	27.92(1.69)
Screen time (min/day) ^a^	411	18.55(2.44)	411	34.95(3.31)	411	35.24(3.27)
Sleep time (min/day) ^a^	407	830.43(8.98)	407	833.98(7.72)	407	818.00(6.21)
ASQ-3 Communication (Range: 0–60) ^a^	411	47.39(0.55)	411	52.04(0.41)	411	49.63(0.48)
ASQ-3 Fine motor (Range: 0–60) ^a^	411	45.58(0.47)	411	50.81(0.55)	411	46.49(0.70)
ASQ-3 Gross motor (Range: 0–60) ^a^	411	53.61(0.42)	411	54.12(0.42)	411	42.73(0.65)
ASQ-3 Personal-social (Range: 0–60) ^a^	411	49.99(0.41)	411	50.00(0.53)	411	48.55(0.56)
ASQ-3 Problem solving (Range: 0–60) ^a^	411	45.92(0.57)	411	53.52(0.52)	411	49.37(0.61)
ASQ-3 Total (Range: 0–300) ^a^	411	242.49(1.61)	411	260.58(1.62)	411	237.11(1.94)
AIMS Prone (Range: 0–21)	–	–	–	–	125	9.70 (2.71)
AIMS Supine (Range: 0–9)	–	–	–	–	125	7.57(1.16)
AIMS Sitting (Range: 0–12)	–	–	–	–	125	6.20(2.23)
AIMS Standing (Range: 0–16)	–	–	–	–	125	2.67(0.49)
AIMS Total (Range: 0–58) ^b^	–	–	–	–	125	26.14 (5.23)
AIMS Percentile (Range: 0–100) ^b^	–	–	–	–	125	37.19 (26.66)
Milestone independent sitting age (days) ^b^	229	186.26 (26.46)				
Milestone crawling age (days) ^b^	230	253.51 (43.43)				
Milestone assisted standing age (days) ^b^	233	251.17 (42.23)				
Milestone assisted walking age (days) ^b^	233	288.30 (48.71)				
Milestone independent standing age (days) ^b^	231	333.77 (54.86)				
Milestone independent walking age (days) ^b^	244	376.37 (58.36)				

^a^Data presented as estimated marginal mean (standard error)

^b^Data presented as mean (standard deviations)

*Abbreviation*: *ASQ* Age and stage questionnaire, *AIMS* Alberta Infant Motor Scale

The longitudinal associations between movement behaviours and ASQ-3 outcomes are presented in Table 3. Higher tummy time across time points was significantly associated with a higher gross motor (B = 0.19, 95%CI: 0.11,0.27) and personal-social (B = 0.09, 95%CI: 0.02,0.17) development scores over time. Higher reading time across time points was significantly associated with higher ASQ-3 fine motor (B = 0.25, 95%CI: 0.01,0.49), gross motor (B = 0.31, 95%CI: 0.11,0.52), personal-social (B = 0.23, 95%CI: 0.02,0.44), and total development (B = 1.29; 95%CI: 0.54,2.03) scores over time. No other significant associations were observed. In sensitivity analyses among those with valid movement behaviour data at all time points (*n* = 251–254 depending on the outcome), similar findings were observed, except the association between reading and personal-social development was no longer significant. Whereas, a significant negative association between screen time and the problem solving development score was observed (data not shown).

**Table 3:** Longitudinal associations between movement behaviours and ASQ-3 outcomes

	n	Communication ^a^		Fine motor^b^		Gross motor^b^		Personal-social^b^		Problem solving ^a^		Total^c^	
		B (95%CI)	*P* value	B (95%CI)	*P* value	B (95%CI)	*P* value	B (95%CI)	*P* value	B (95%CI)	*P* value	B (95%CI)	*P* value
Tummy time (10 min/day)	411	-0.04 (−0.12,0.03)	0.269	0.07 (− 0.02,0.16)	0.123	**0.19 (0.11,0.27)**	**< 0.001**	**0.09 (0.02,0.17)**	**0.016**	− 0.003 (− 0.09,0.09)	0.954	0.08 (− 0.19,0.35)	0.550
Back time (10 min/day)	410	−0.001 (− 0.04,0.04)	0.977	0.02 (− 0.02,0.07)	0.336	− 0.02 (− 0.06,0.02)	0.255	−0.03 (− 0.07,0.01)	0.178	0.01 (− 0.04,0.06)	0.667	0.07 (− 0.08,0.21)	0.363
Restrained time (10 min/day)	411	−0.001 (− 0.05,0.05)	0.973	− 0.02 (− 0.07,0.03)	0.507	−0.003 (− 0.05,0.04)	0.908	0.02 (− 0.03,0.06)	0.439	−0.03 (− 0.08,0.02)	0.252	−0.01 (− 0.17,0.16)	0.929
Screen time (10 min/day)	411	0.05 (−0.06,0.17)	0.378	−0.02 (− 0.14,0.10)	0.754	0.11 (− 0.003,0.21)	0.057	− 0.07 (− 0.18,0.04)	0.200	− 0.10 (− 0.23,0.02)	0.113	0.10 (− 0.29,0.50)	0.605
Reading time (10 min/day)	411	0.15 (− 0.07,0.37)	0.182	**0.25 (0.01,0.49)**	**0.040**	**0.31 (0.11,0.52)**	**0.003**	**0.23 (0.02,0.44)**	**0.028**	0.21 (−0.03,0.46)	0.087	**1.29 (0.54,2.03)**	**0.001**
Sleep time (hour/day)	407	−0.04 (− 0.29,0.20)	0.725	− 0.04 (− 0.31,0.22)	0.737	0.14 (− 0.09,0.36)	0.224	− 0.05 (− 0.28,0.18)	0.663	−0.04 (− 0.31,0.24)	0.790	0.09 (− 0.77,0.94)	0.840

Participants who had at least one observations on variables of interest were include in the analyses. In all models, covariates (Baseline: infant sex, infant race/ethnicity, number of siblings, parental marital status, parental education, parental country of birth; Time varying: non-parental care time, parental age) were included as fixed effects. Mean imputation was performed for missing parental age at baseline (*n* = 5)

^a^Models include a random intercept, a fixed linear slope of infant age (centered), and a fixed quadratic linear slope of infant age (centered)

^b^Models include a random intercept, a random slope of slope of infant age (centered), a fixed linear slope of infant age (centered), and a fixed quadratic linear slope of infant age (centered)

^c^Models include a random intercept and a fixed linear slope of infant age (centered)

**Bold fonts** indicate *p* < 0.05

The longitudinal associations between average movement behaviours across the three time points and milestone age outcomes are presented in Table 4. Higher tummy time was significantly associated with earlier acquisition of all six major gross motor milestones (independent sitting: B = -0.74, 95%CI: − 1.42,-0.06; crawling: B = -3.32, 95%CI: − 4.40,-2.24; assisted standing: B = -2.18, 95%CI: − 3.32,-1.04; assisted walking: B = -2.58, 95%CI: − 3.83,-1.33; independent standing: B = -3.22, 95%CI: − 4.58,-1.86, independent walking: B = -3.41, 95%CI:-4.85,-1.98). In contrast, higher back time across time points was significantly associated with a later acquisition of assisted standing (B = 0.86, 95%CI: 0.24,1.47), assisted walking (B = 0.74, 95%CI: 0.02,1.46), and independent walking (B = 0.82, 95%CI: 0.01,1.62). Similarly, higher restrained time across time points was significantly associated with a later acquisition of supported walking (B = 0.89, 95%CI:0.11,1.66). No other significant associations were observed. In sensitivity analyses among those with valid movement behaviour data at all time points (*n* = 205–219), similar findings were observed, except the association between back time and independent walking was no longer significant (data not shown). In additional sensitivity analyses among those where exact milestone acquisition dates were reported (*n* = 197–226 or 85–93%), similar findings were observed, except higher screen time was significantly associated with earlier acquisition of independent sitting (see Table S2).

**Table 4:** Longitudinal associations between average movement behaviours across the three time points and milestone age outcomes

	Independent sitting (*n* = 229)		Crawling (*n* = 230)		Assisted standing (*n* = 233)		Assisted walking (*n* = 233)		Independent standing (*n* = 231)		Independent walking (*n* = 244)	
	B (95%CI)	*P* value	B (95%CI)	*P* value	B (95%CI)	*P* value	B (95%CI)	*P* value	B (95%CI)	*P* value	B (95%CI)	*P* value
Tummy time (10 min/day)	**−0.74 (− 1.42,-0.06)**	**0.034**	**−3.32 (− 4.40,-2.24)**	**< 0.001**	**− 2.18 (− 3.32,-1.04)**	**< 0.001**	**− 2.58 (− 3.83,-1.33)**	**< 0.001**	**− 3.22 (− 4.58,-1.86)**	**< 0.001**	**−3.41 (− 4.85,-1.98)**	**< 0.001**
Back time (10 min/day)	0.16 (− 0.20,0.52)	0.390	0.25 (− 0.36,0.85)	0.425	**0.86 (0.24,1.47)**	**0.006**	**0.74 (0.02,1.46)**	**0.043**	0.55 (−0.23,1.32)	0.169	**0.82 (0.01,1.62)**	**0.046**
Restrained time (10 min/day)	0.14 (−0.27,0.55)	0.504	0.25 (−0.42,0.92)	0.466	0.51 (−0.15,1.17)	0.132	**0.89 (0.11,1.66)**	**0.025**	0.10 (−0.74,0.95)	0.809	0.46 (−0.43,1.34)	0.314
Screen time (10 min/day)	−0.80 (− 1.75,0.15)	0.097	− 0.90 (−2.48,0.68)	0.262	−1.12 (− 2.63,0.39)	0.147	− 1.10 (− 2.88,0.69)	0.228	− 1.82 (−3.80,0.16)	0.072	− 0.77 (− 2.88,1.33)	0.472
Reading time (10 min/day)	0.04 (− 1.80,1.89)	0.962	− 0.01 (− 2.83,2.85)	0.995	0.74 (− 1.98,3.46)	0.594	− 0.87 (− 4.04,2.30)	0.537	0.38 (− 3.22,4.00)	0.835	− 1.09 (−4.95,2.78)	0.582
Sleep time (hour/day)	0.28 (− 1.74,2.30)	0.784	0.81 (− 2.51,4.14)	0.632	1.10 (− 1.97,4.16)	0.484	1.88 (− 1.79,5.55)	0.316	2.28 (− 1.92,6.48)	0.288	2.54 (− 1.67,6.75)	0.236

**Bold fonts** indicate *p* < 0.05

Results are pooled for all imputation data sets. Covariates (Baseline: infant sex, infant race/ethnicity, number of siblings, parental age, parental marital status, parental education, parental country of birth; Average across time points: non-parental care time) were included in all models. Mean imputation was performed for missing parental age at baseline (*n* = 1)

*Abbreviations*: *B* Unstandardized beta coefficient, *CI* Confidence interval

Longitudinal associations between average movement behaviours across the three time-points and AIMS outcomes are presented in Table 5. Higher tummy time across time points was associated with significantly higher prone (B = 0.16, 95%CI: 0.06,0.27), total (B = 0.30, 95%CI: 0.11,0.50) and percentile (B = 1.64, 95%CI: 0.63,2.64) AIMS scores at 6 months. Whereas, higher back time across the three time points was associated with significantly lower prone (B = -0.08, 95%CI: − 0.14,-0.01), supine (B = -0.04, 95%CI: − 0.06,0.01), total (B = -0.14, 95%CI: − 0.26,-0.03), and percentile (B = -0.67: 95%CI: − 1.26,-0.09) AIMS scores. Higher restrained time (OR = 0.91, 95%CI: 0.86,0.96) and screen time (OR = 0.83, 95%CI: 0.72,0.95) across the three time points were significantly associated with a lower likelihood of being in the group with the higher stand AIMS scores. No other significant association was observed. In sensitivity analyses among those with valid movement behaviour data at all time points (*n* = 109), similar findings were observed, except higher restrained time was significantly associated with lower supine and percentile AIMS scores (data not shown).

**Table 5:** Longitudinal associations between average movement behaviours across the three time-points and AIMS outcomes

	n	Prone		Supine		Sit		Stand^1^		Total		Percentile	
		B (95%CI)	*P* value	B (95%CI)	*P* value	B (95%CI)	*P* value	OR (95%CI)	*P* value	B (95%CI)	*P* value	B (95%CI)	*P* value
Tummy time (10 min/day)	125	**0.16 (0.06,0.27)**	**0.002**	0.04 (− 0.001,0.08)	0.058	0.08 (− 0.01,0.16)	0.070	1.11 (1.00,1.23)	0.054	**0.30 (0.11,0.50)**	**0.003**	**1.64 (0.63,2.64)**	**0.001**
Back time (10 min/day)	125	**− 0.08 (− 0.14,-0.01)**	**0.022**	**− 0.04 (− 0.06, -0.01)**	**0.007**	− 0.02 (− 0.07, 0.02)	0.324	0.96 (0.92,1.00)	0.077	**− 0.14 (− 0.26,-0.03)**	**0.015**	**−0.67 (−1.26,-0.09)**	**0.023**
Restrained time (10 min/day)	125	−0.04 (− 0.10,0.02)	0.218	− 0.03 (− 0.05,0.001)	0.057	−0.02 (- 0.07,0.03)	0.483	**0.91 (0.86,0.96)**	**0.001**	−0.09 (− 0.22,0.02)	0.086	− 0.54 (−1.15,0.06)	0.077
Screen time (10 min/day)	125	−0.05 (− 0.21,0.12)	0.587	− 0.03 (− 0.10,0.04)	0.366	−0.02 (− 0.15,0.10)	0.724	**0.83 (0.72,0.95)**	**0.007**	−0.14 (− 0.44,0.17)	0.369	−0.88 (− 2.42,0.67)	0.266
Reading time (10 min/day)	125	0.05 (−0.25,0.35)	0.755	−0.06 (− 0.19,0.06)	0.322	0.10 (− 0.13,0.33)	0.401	1.02 (0.82,1.28)	0.830	0.09 (−0.46,0.65)	0.741	0.70 (− 2.11,3.50)	0.627
Sleep time (hour/day)	125	0.13 (−0.21,0.46)	0.458	−0.04 (− 0.19,0.10)	0.559	(− 0.28,0.25)	0.927	0.92 (0.71,1.19)	0.504	0.05 (−0.60,0.69)	0.887	−0.07 (−3.32,3.19)	0.968

**Bold fonts** indicate *p* < 0.05

^1^Due to distribution, AIMS stand was dichotomised based on median as a dummy variable (value = 1 [score = 2, reference group]; value = 0 [score > 2])

Results are pooled for all imputation data sets. Covariates (AIMS assessment: infant age; Baseline: infant sex, infant race/ethnicity, number of siblings, parental age, parental marital status, parental education, parental country of birth; Average across time points: non-parental care time) were included in all models. Mean imputation was performed for missing parental age at baseline (*n* = 1)

*Abbreviations*: *B* Unstandardized beta coefficient, *CI* Confidence interval, *ASQ* Age and stage questionnaire

## Discussion

This novel study addressed gaps and limitations in the evidence base by examining the longitudinal associations of physical activity, sedentary behaviour, and sleep with physical, social-emotional, and cognitive development in a large and diverse sample of typically developing infants. In support of our first hypothesis, tummy time was consistently associated with more advanced gross motor development over time, including the earlier acquisition of gross motor milestones. In partial support of our second hypothesis, some longitudinal associations were also observed between higher restrained time and less advanced gross motor development, especially when considering sensitivity analyses, though a number of null associations were also observed. Similar findings were observed for higher back time. In contrast, higher reading was associated with more advanced total development over time. Generally, observed associations had small effect sizes.

National and international guidelines recognize that tummy time is an important type of physical activity in non-mobile infants [1, 2]. Specifically, it is recommended that infants who are not yet mobile should accumulate at least 30 min/day of tummy time throughout waking periods [1, 2]. Our finding that tummy time was consistently associated with more advanced gross more development aligns with the findings of two previous systematic reviews [3, 8], including one that informed this tummy time recommendation [3]. Our findings also build on these review findings [3, 8] by addressing limitations in the previous literature related to study design, sample size, selection bias, performance bias, and residual confounding. Tummy time is thought to be particularly important for motor development in infants because it supports head control and anti-gravity extension of the trunk, and as infants develop, tummy time can improve stability in weight-bearing positions, such as sitting and prone on hands and knees [9]. Apart from motor development, we did not observe any other consistent associations between tummy time and other development measures. This finding is in line with conclusions based on a small number of included studies in the previously mentioned systematic review [8], though our findings were not consistent with one included longitudinal study in a large sample of infants (*n* = 1804) from Japan who also used the ASQ-3 tool [28]. Specifically, infants in the Japanese study who could stay prone on extended arms at 6 months had significantly higher ASQ-3 communication, fine motor, problem solving, personal-social, and total development scores at 6 months, compared to infants who could not [28]. These differences persisted to 1–2 years of age, depending on the outcome, though after 1.5 years effect sizes were small [28]. The differences in findings with the present study may be explained by the fact we measured tummy time duration across the first 6 months of life versus tummy time ability at 6 months. These measurements are fundamentally different as tummy time for younger infants represents the amount of time they are placed in prone compared to the ability to maintain extended arm support in prone, an indicator of infant gross motor ability. Overall, our findings support the promotion of tummy time in the first 6 months of life.

A novel aspect of the present study was the comprehensive examination of multiple types of sedentary behaviour. Observed associations between the different types of sedentary behaviour and outcome variables were not all in the same direction. For instance, increased restrained and back time tended to be associated with less advanced gross motor development, whereas reading time was associated with more advanced total development. Additionally, the associations of screen time were primarily null. This is in line with national and international guidelines that discourage some sedentary behaviours (i.e., prolonged restrained time, screen time) and encourage others (e.g., reading) [1, 2]. The evidence for some types of sedentary behaviour, including back time, restrained time, and reading is extremely limited [4]. In fact, no single study included in the systematic reviews that informed the national and international sedentary behaviour recommendations included all the sedentary behaviour types examined in the present study [4]. The study confirms the importance of measuring specific types of sedentary behaviour versus total sedentary time in future research examining associations with development in infants.

In contrast to other movement behaviours, sleep time was not related to infant development in the first 6 months. This finding is consistent with the mixed or null associations observed in previous studies focusing on infant sleep time. Specifically, in a previous systematic review examining the associations between sleep time and health indicators, there were inconsistent associations between sleep time and emotional regulation (positive: *n* = 3; null: *n* = 4; negative: *n* = 1) and null associations between sleep time, cognitive development (null: *n* = 4), and motor development (*n* = 2) across studies with infant samples [29]. In particular, two cross-sectional studies included in this review that measured development with the ASQ-3 tool found no significant associations for sleep time among infants [30, 31]. In these two studies, sleep time was measured using a questionnaire in a sample of 1351 Brazilian children aged 3 to 13 months [30] and with an accelerometer in a sample of 52 New Zealander children aged 11–13 months [31]. However, these null findings for sleep time do not negate the importance of sleep for infant development, as other sleep dimensions may play a role. For instance, positive associations for nighttime efficiency (with fine motor and problem-solving skills) and percentage of sleep time at night (with communication and problem-solving skills) were observed in regard to ASQ-3 outcomes in the New Zealander sample [31]. Therefore, future research examining multiple sleep dimensions (e.g., sleep time, efficacy, fragmentation, latency, problems) is needed to further understand the relationship between sleep and development during infancy, particularly in the first 6 months.

This study has made novel contributions to the evidence base. Given the limited evidence in this area, there remain a number of directions for future research. First, the present study conducted separate models for each movement behaviour and development outcome because these movement behaviours are not independent of each other [32]. Recently, compositional analysis has been used in older age groups to examine the combined associations of mutually exclusive and co-dependent movement behaviours with health indicators [32]. In our sample, not all movement behaviours measured via the questionnaire were mutually exclusive. For example, screen time or reading could have occurred at the same time as restrained time or back time. Therefore, future research examining the association between movement behaviours and development in infants should use data collection procedures (e.g., accelerometer, time-use diary) and data analysis methods that can appropriately examine these combined associations. Second, national and international guidelines include a recommendation that infants should not be restrained for prolonged periods, defined as more than 1 h [1, 2]. The questionnaire in the Early Movers study did not capture bouts of restrained time, instead it captured total duration. Therefore, future research should examine the association of this prolonged dose of restrained time, along with the associations between other doses of movement behaviours and development. Finally, the study followed children during the first 18 months of life, future research is needed to examine the association between infant movement behaviours and development outcomes throughout early childhood and beyond.

This study has a number of strengths that addressed previous limitations in the evidence base. These strengths include the focus on an understudied age group, the longitudinal design, the relatively large and diverse sample, the adjustment for various confounders, the inclusion of a variety of movement behaviours, especially various types of sedentary behaviour, with acceptable psychometric properties, and the inclusion of a variety of developmental outcomes with acceptable psychometric properties that span different developmental domains. Given these strengths, findings may be generalizable to infants in Alberta or across Canada. In terms of limitations, though our measures had acceptable psychometric properties and were feasible to administer in a large and diverse sample, most were parental-reported and therefore more susceptible to recall and social desirability bias. Additionally, even though we adjusted for a number of covariates, residual confounding may still have occurred.

## Conclusions

In a large and diverse sample of typically developing infants, tummy time was consistently longitudinally associated with more advanced gross motor development, and reading was longitudinally associated with more advanced overall development over time. In contrast, back and restrained time were associated with less advanced gross motor development for some outcomes. These specific types of physical activity and sedentary behaviour appear important to target in future interventions and initiatives. Future research should consider compositional approaches, different doses of movement behaviours, as well as longer-term implications of infant movement behaviours.

## Supplementary Information

**Additional file 1 Supplementary Table 1.** Spearman’s rank correlations between questionnaire and time-use diary measures of movement behaviours across three time points. **Supplementary Table 2.** Longitudinal associations between average movement behaviours across the three time points and exact milestone age outcomes.

## Data Availability

The dataset generated and analysed during the current study are not publicly available due ethical restrictions but are available from the corresponding author on reasonable request.

## Abbreviations

AIMS: Alberta Infant Motor Scale
ASQ-3: Ages & Stages Questionnaire
B: Unstandardized beta coefficient
CI: Confidence Interval
WHO: World Health Organization
