# Supplementary material for: Longitudinal associations between infant movement behaviours and development
Source: Int J Behav Nutr Phys Act. 2022 Jan 28;19:10. doi: 10.1186/s12966-022-01248-6 (PMC8800227; doi:10.1186/s12966-022-01248-6)
Supplement: Supplementary file 1 — Additional file 1 Supplementary Table 1. Spearman’s rank correlations between questionnaire and time-use diary measures of movement behaviours across three time points. Supplementary Table 2. Longitudinal associations between average movement behaviours across the three time points and exact milestone age outcomes. [file 12966_2022_1248_MOESM1_ESM.docx]

**Table S1.** Spearman’s rank correlations between questionnaire and time-use diary measures of movement behaviours across three time points

|  | 2 months (n=121) | | 4 months (n=102) | | 6 months (n=99) | |
| --- | --- | --- | --- | --- | --- | --- |
|  | r_s_ | P value | r_s_ | P value | r_s_ | P value |
| Tummy time | 0.30 | 0.001 | 0.49 | <0.001 | 0.50 | <0.001 |
| Restrained time | 0.32 | <0.001 | 0.37 | <0.001 | 0.31 | 0.005 |
| Reading time | 0.36 | <0.001 | 0.50 | <0..001 | 0.40 | <0.001 |
| Screen time | 0.36 | <0.001 | 0.55 | <0.001 | 0.50 | <0.001 |
| Sleep time | 0.39 | <0.001 | 0.36 | <0.001 | 0.56 | <0.001 |

**Table S2.** Longitudinal associations between average movement behaviours across the three time points and exact milestone age outcomes

|  | Independent sitting (n=208) | | Crawling  (n=212) | | Assisted standing  (n=208) | | Assisted walking  (n=197) | | Independent standing  (n=197) | | Independent walking  (n=226) | |
| --- | --- | --- | --- | --- | --- | --- | --- | --- | --- | --- | --- | --- |
|  | B  (95%CI) | P value | B  (95%CI) | P value | B  (95%CI) | P value | OR  (95%CI) | P value | B  (95%CI) | P value | B  (95%CI) | P value |
| Tummy time (10min/day) | **-0.83**  **(-1.56,-0.09)** | **0.027** | **-3.39**  **(-4.50,-2.27)** | **<0.001** | **-2.25**  **(-3.45,-1.05)** | **<0.001** | **-2.55**  **(-3.86,-1.24)** | **<0.001** | **-2.94**  **(-4.37,-1.51)** | **<0.001** | **-3.65**  **(-5.12,-2.18)** | **<0.001** |
| Back time (10min/day) | 0.35  (-0.03,0.73) | 0.074 | 0.35  (-0.29,0.98) | 0.286 | **1.13**  **(0.48,1.77)** | **0.001** | **0.87**  **(0.10,1.64)** | **0.027** | 0.74  (-0.11,1.59) | 0.086 | **0.85**  **(0.02,1.69)** | **0.045** |
| Restrained time  (10min/day) | 0.15  (-0.27,0.57) | 0.490 | 0.27  (-0.42,0.96) | 0.445 | 0.38  (-0.31,1.07) | 0.277 | **0.89**  **(0.08,1.69)** | **0.032** | 0.11  (-0.80,1.02) | 0.811 | 0.40  (-0.52,1.32) | 0.392 |
| Screen time (10min/day) | **-0.99**  **(-1.96,-0.02)** | **0.045** | -0.99  (-2.62,0.63) | 0.231 | -1.28  (-2.83,0.27) | 0.106 | -0.93  (-2.79,0.93) | 0.325 | -1.49  (-3.54,0.56) | 0.155 | -0.76  (-2.95,1.43) | 0.496 |
| Reading time (10min/day) | 0.21  (-1.86,2.28) | 0.843 | -0.55  (-3.84,2.74) | 0.742 | 0.21  (-2.95,3.36) | 0.898 | -0.27  (-3.95,3.42) | 0.888 | 1.98  (-1.81,5.77) | 0.306 | -0.90  (-4.85,3.04) | 0.656 |
| Sleep time (hour/day) | -1.19  (-3.40,1.02) | 0.292 | 1.68  (-1.80,5.17) | 0.344 | 1.17  (-2.12,4.45) | 0.487 | 2.36  (-1.59,6.31) | 0.241 | 3.29  (-1.36,7.93) | 0.165 | 3.12  (-1.23,7.46) | 0.159 |

Abbreviations: B, unstandardized beta coefficient; CI, confidence interval

**Bold fonts** indicate p<0.05.

Note: Results are pooled for all imputation data sets. Covariates (Baseline: infant age, infant sex, infant race/ethnicity, number of siblings, parental age, parental marital status, parental education, parental country of birth; Average across time points: non-parental care time) were included in all models. Mean imputation was performed for missing parental age at baseline (n=1).
